# Fitness and transcriptomic analysis of pathogenic *Vibrio parahaemolyticus* in seawater at different shellfish harvesting temperatures

**DOI:** 10.1128/spectrum.02783-23

**Published:** 2023-11-14

**Authors:** Zhuosheng Liu, Chao Liao, Luxin Wang

**Affiliations:** 1 Department of Food Science and Technology, University of California, Davis, California, USA; The Pennsylvania State University, University Park, Pennsylvania, USA

**Keywords:** *Vibrio parahaemolyticus*, seawater, *tdh*, *trh*, survival and growth models, RNA-seq

## Abstract

To better characterize the population dynamics of *Vibrio parahaemolyticus* (*Vp*) containing different virulence genes, two *Vp* strains were inoculated into seawater separately and incubated at temperatures (10°C and 30°C) mimicking winter and summer pre-harvest shellfish rearing seasons. The cellular responses of two *Vp* strains, one containing the thermostable direct hemolysin (*tdh*+) gene and the other one containing *tdh*-related hemolysin (*trh*+) gene, were studied at the transcriptomic level. Results showed that, at 30°C, *tdh*+ and *trh*+ strains reached 6.77 ± 0.20 and 6.14 ± 0.07 log CFU/mL, respectively, after 5 days. During this time, a higher growth rate was observed in the *tdh*+ strain than in the *trh*+ strain. When being kept at 10°C, both *Vp* strains persisted at ca. 3.0 log CFU/mL in seawater with no difference observed between them. Survival and growth models were then established based on the Baranyi equation. The goodness-of-fit scores ranged from 0.674 to 0.950. RNA sequencing results showed that downregulated central energy metabolism and weakened degradation of branched-chain amino acid were observed only in the *trh*+ strain not in the *tdh*+ strain at 30°C. This might be one reason for the lower growth rates of the *trh*+ strain at 30°C. Histidine metabolism and biofilm formation pathways were significantly downregulated in both strains at 10°C. No significant difference was observed for virulence-associated gene expression between 10°C and 30°C, regardless of the strains.

**IMPORTANCE:**

Given the involvement of *Vibrio parahaemolyticus* (*Vp*) in a wide range of seafood outbreaks, a systematical characterization of *Vp* fitness and transcriptomic changes at temperatures of critical importance for seafood production and storage is needed. In this study, one of each virulent *Vp* strain (*tdh*+ and *trh*+) was tested. While no difference in survival behavior of the two virulent strains was observed at 10°C, the *tdh*+ strain had a faster growth rate than the *trh*+ strain at 30°C. Transcriptomic analysis showed that a significantly higher number of genes were upregulated at 30°C than at 10°C. The majority of differentially expressed genes of *Vp* at 30°C were annotated to functional categories supporting cellular growth. At 10°C, the downregulation of the biofilm formation and histidine metabolism indicates that the current practice of storing seafood at low temperatures not only protects seafood quality but also ensures seafood safety.

## INTRODUCTION

*Vibrio parahaemolyticus* (*Vp*) has been a leading seafood-borne pathogenic bacterium (Gram negative, curved rod shaped) commonly found in marine environments, particularly in estuaries and coastal waters; it has been a major microbiological food safety concern for aquacultural products, especially raw oysters (1). To mitigate the risk of *Vp* contamination, shellfish farmers follow guidelines such as the National Shellfish Sanitation Program in the United States for harvesting, handling, and storing oysters (2). Despite these control measures, over 36,000 *Vp* infection cases associated with shellfish were annually reported in the United States, and recalls associated with *Vp* contaminated seafood products continue to occur (3 – 6). The undesirable public health consequences of *Vp*-contaminated food also bring economic burden. For instance, the estimated total cost of illness associated with *Vp* infection increased from $40,682,312 in 2013 to $45,735,332 in 2018 in the United States (7).

Given its widely reported prevalence in seafood farming environment and seafood products, efforts have been made to characterize behaviors of *Vp* in different stages from sea to folk (8, 9). Among different environmental parameters, temperature is one dominant factor significantly impacting the behavior of *Vp* (10). In coastal environment, significantly lower levels of *Vp* or prevalence have been reported in winter months than summer (11, 12). This common finding was also accompanied by lower *Vp* infection incidence rate in colder months based on the National Outbreak Reporting System (13). This real-world evidence, taken together, underscores the correlation between ambient temperature and *Vp* prevalence and incidence of *Vp*-related Vibriosis.

RNA sequencing (RNA-seq) is a next-generation transcriptomic technique illuminating gene expression patterns in targeted organism, which can be applied to yield biological insights about physiological state of bacterial pathogens under different conditions (14, 15). Transcriptomic analysis has been used to investigate essential cellular mechanisms of *Vp* when surviving under simulated post-harvest practices (PHPs) (e.g., cold storage, high-salinity relaying, and acid-driven PHPs) (16 – 20). However, the physiological changes of *Vp* in natural seafood production environment, the impact of different virulence genes on its behavior, and how pre-harvest environment impacts the behavior of *Vp* during post-harvest handling and processing remain largely unknown. Therefore, this study aims to better understand the persisting mechanism of *Vp* in natural shellfish rearing environment, particularly its cellular responses and virulence. This information, in turn, can support the development of novel control and monitoring strategies. The specific aims of this study were (i) investigating the survival and growth of *Vp* with different virulence genes in seawater at 10°C and 30°C and establishing survival and growth models for predicting *Vp* population under different conditions and (ii) profiling gene expression of *Vp* at 10°C and 30°C and identifying key changes in metabolic pathways and virulence factors.

## RESULTS AND DISCUSSION

### Primary models predicting *Vp* fitness in seawater at different harvesting temperatures

**Fig 1:**
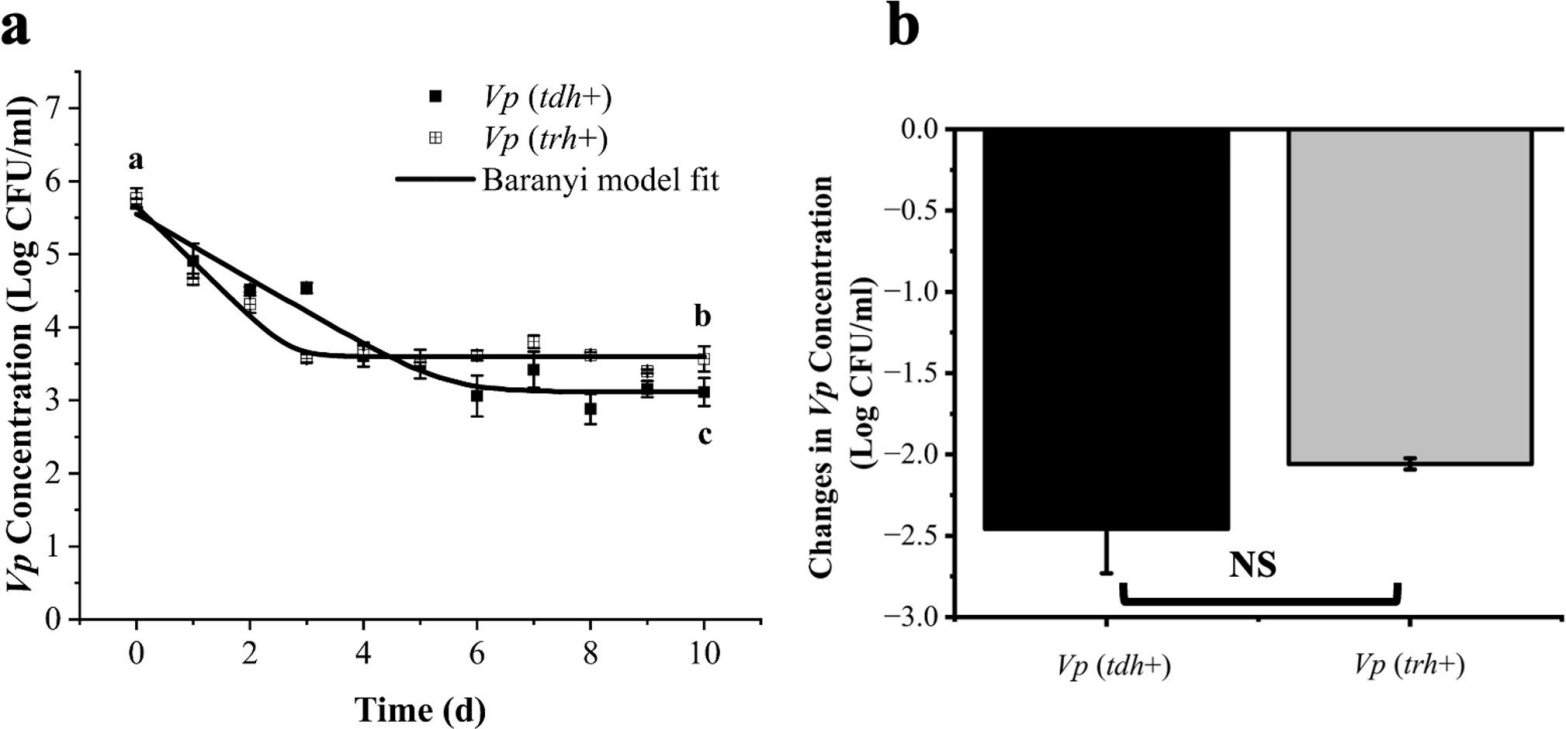
Survival models of pathogenic *V. parahaemolyticus* strains (ATCC 43996 *tdh*+ and ATCC 17802 *trh*+) kept in seawater at 10°C for 10 days (**a**), and the comparison of the cell concentration reductions of two strains on Day 10 (**b**). The Baranyi model was used to fit the enumeration data of *Vp*. Different lower case letters represent significant differences in bacterial counts between the *tdh*+ and the *trh*+ strains at different sampling points. NS indicates that no significant difference was observed.

**Fig 2:**
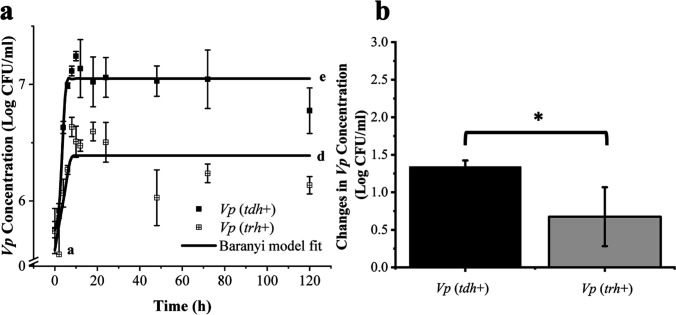
Growth models of pathogenic *V. parahaemolyticus* strains (ATCC 43996 *tdh*+ and ATCC 43996 *trh*+) growing in seawater at 30°C for 120 hours (5 days) (**a**), and the comparison of the cell concentration increases of two strains (**b**). The Baranyi model was used to fit the enumeration data of *Vp*. Different lower case letters represent significant differences in bacterial count between *tdh*+ and *trh*+ strains at different sampling points. Asterisk (*) represents a significant difference (*P* < 0.05).

The pH and salinity levels of the used seawater collected from oyster research farm were 7.82% and 2.0%, respectively, both within the typical range found in natural coastal environments (21, 22). Populations of *Vp* stored in seawater at 10°C or 30°C over 10 or 5 days were enumerated based on the plate count method (Fig. 1 and 2). For the 10°C trials, the inoculation level of *Vp* in seawater was 5.70 ± 0.06 and 5.76 ± 0.14 log CFU/mL for *tdh*+ [American Type Culture Collection (ATCC) 43996] and *trh*+ (ATCC 17802) strains, respectively. More rapid population decrease was observed in the *trh*+ strain compared to the *tdh*+ strain. Continuous decreases of culturable *Vp* cells (ca. 2.0 log CFU/mL) were observed from Day 0 to Day 7, and populational levels reached 3.11 ± 0.19 and 3.57 ± 0.17 log CFU/mL for *tdh*+ and *trh*+, respectively, on Day 10. For the 30°C storage trial, the initial inoculation level of *Vp* in seawater was 5.75 ± 0.07 and 5.74 ± 0.19 log CFU/mL for *tdh*+ and *trh*+ strains, respectively. Growth of *tdh*+ and *trh*+ strains was observed, and both strains reached the stationary phase with population levels at 7.11 ± 0.04 and 6.64 ± 0.08 log CFU/mL, respectively, after 8 hours. After *Vp* reaching the stationary phase, significantly higher populational level in *tdh*+ compared with *trh*+ persisted throughout the rest of incubation time. Difference in fitness between *Vp* strains containing different virulence genes has been reported by previous studies. Khouadja et al. (23) reported that *tdh*+ strain showed higher growth rate compared with *trh* strain when inoculated in sea bass serum and stored at 30°C for 240 min.

The survival and growth data of *Vp* in seawater at 10°C and 30°C obtained from plate count results were further fitted by the Baranyi function to establish survival and growth models (Fig. 1 and 2). The detailed parameters of Baranyi-based models predicting *Vp* fitness in seawater are listed in Table 1. The *R* ^2^ of Baranyi models of *Vp* in seawater at 10°C were 0.94 and 0.96 for *tdh*+ and *trh*+ strains, respectively. At 10°C, populations of the *Vp tdh*+ strain decreased from the initial values (IVs) to the final values (FVs) by 2.44 log CFU/mL; meanwhile, the *trh* strain decreased from the IVs to the FVs by 2.06 log CFU/mL. The specific inactivation rate (SIR) in seawater at 10°C was −0.45 ± 0.060 and −0.76 ± 0.11 log CFU/d for *tdh*+ and *trh*+ strains, respectively. No significant difference in *Vp* population reduction over 10 days at 10°C was observed between *tdh*+ and *trh*+ strains based on difference between model predicted initial value and final value. Liao et al. (24) stored oysters inoculated with a five-strain *Vp* cocktail at 4.69 log CFU/g at 10°C for 11 days and reported SIR values of −0.073 ± 0.017 log CFU/day. The difference in SIR values between the current study and the previous study might be caused by the different nutrient levels available in oysters vs in seawater.

**TABLE 1:** Survival and growth models predicting *Vibrio parahaemolyticus* fitness in seawater at 10°C and 30°C^*a*^

Temperature	Parameters	*Vp* (ATCC 43996 *tdh*+)	*Vp* (ATCC 17802 *trh*+)
10°C	*R* ^2^	0.943	0.95
	IV (log CFU/mL)	5.554 ± 0.164	5.655 ± 0.143
	FV (log CFU/mL)	3.115 ± 0.108	3.6 ± 0.058
	SIR (log CFU/d)	−0.447 ± 0.0602	−0.758 ± 0.111
30°C	*R* ^2^	0.931	0.674
	IV (log CFU/mL)	5.751 ± 0.127	5.435 ± 0.185
	FV (log CFU/mL)	7.047 ± 0.0427	6.387 ± 0.0768
	SGR (log CFU/h)	0.391 ± 0.125	0.146 ± 0.0575

^*a*^ IV, initial value of *Vp* population; FV, final value; SIR, specific inactivation rate; SGR, specific growth rate.

When the storage temperature was kept at 30°C, the population of *tdh*+ strain increased from IVs to the FVs with a growth rate of 1.30 log CFU/mL; the *trh+* strain increased from the IVs to the FVs with a growth rate of 0.96 log CFU/mL. The *R* ^2^ of the Baranyi models of *Vp* in seawater at 30°C were 0.97 and 0.95 for *tdh*+ and *trh+* strains, respectively. Higher specific growth rate was observed on *tdh*+ (0.39 ± 0.13 log CFU/d) than *trh*+ strain (0.15 ± 0.058 log CFU/d) (*P* < 0.05).

### Transcriptomic profiles of *Vp* strains when surviving at different temperatures

To investigate the driven factors leading to observed differential behaviors between *tdh*+ and *trh+* strains, transcriptomic changes of each strain were studied by extracting the RNA after 5 days of persistence in seawater at 10°C and 30°C. The transcriptome profile of each strain 2 hours after inoculation into the seawater was used as the control. The transcriptomic analysis procedures were as follows: raw RNA-seq reads were mapped to the reference transcriptome using Salmon. High genome similarity scores were calculated among ATCC 17802, ATCC 43996, and RIMD 2210633, which ranged from 0.986 to 0.987 (Fig. S2). The mapping rate of aligning raw sequence reads with the reference transcriptome ranged from 65.30% to 78.30%. A total of 4,001 genes were successfully identified after the Salmon quasi-mapping against the protein coding sequence of *Vp* RIMD 2210633. The Pearson correlation coefficient (PCC) of *Vp* gene expression profiles in the control (2 hours after seawater inoculation) and the test groups (10°C and 30°C incubation over 5 days) was calculated to examine the linear relationship of gene expression patterns. As shown in Fig. S3a, PCC of *Vp* transcriptome was conducted among three conditions (control reference, 5 days of storage at 10°C, and 5 days of storage at 30°C). The PCC of *Vp* transcriptome between *tdh*+ and *trh*+ strains in the control group was 0.99 suggesting that the gene expression pattern of *tdh*+ and *trh*+ strains before storage was similar and could serve as the control reference for following analysis. The PCC of *Vp* transcriptome analysis between the *tdh*+ and the *trh*+ strains after 5-day incubation at 10°C was 0.94, and the PCC of *Vp* transcriptome between *tdh*+ and *trh*+ strains was 0.89 after 5-day incubation at 30°C. This indicated that correlation of gene expression pattern between *tdh*+ and *trh*+ strains was reduced at 30°C compared with 10°C. Principal component analysis (PCA) of *Vp* transcriptomic data was conducted to examine variances in gene expression among samples. Principal component 1 and principal component 2 explained 48.69% and 16.89% variances in gene expression of sequenced transcript reads among control and test groups (Fig. S3b). The transcriptome of *Vp* at 10°C was close to counterparts in control condition, whereas transcriptome of *Vp* at 30°C was separated from counterparts in control condition. There was slightly more variation in *Vp* transcriptome profiles between *tdh*+ and *trh*+ strains at 10°C (*R* ^2^ = 0.67) than 30°C (*R* ^2^ = 0.73) (Fig. S4).

**Fig 3:**
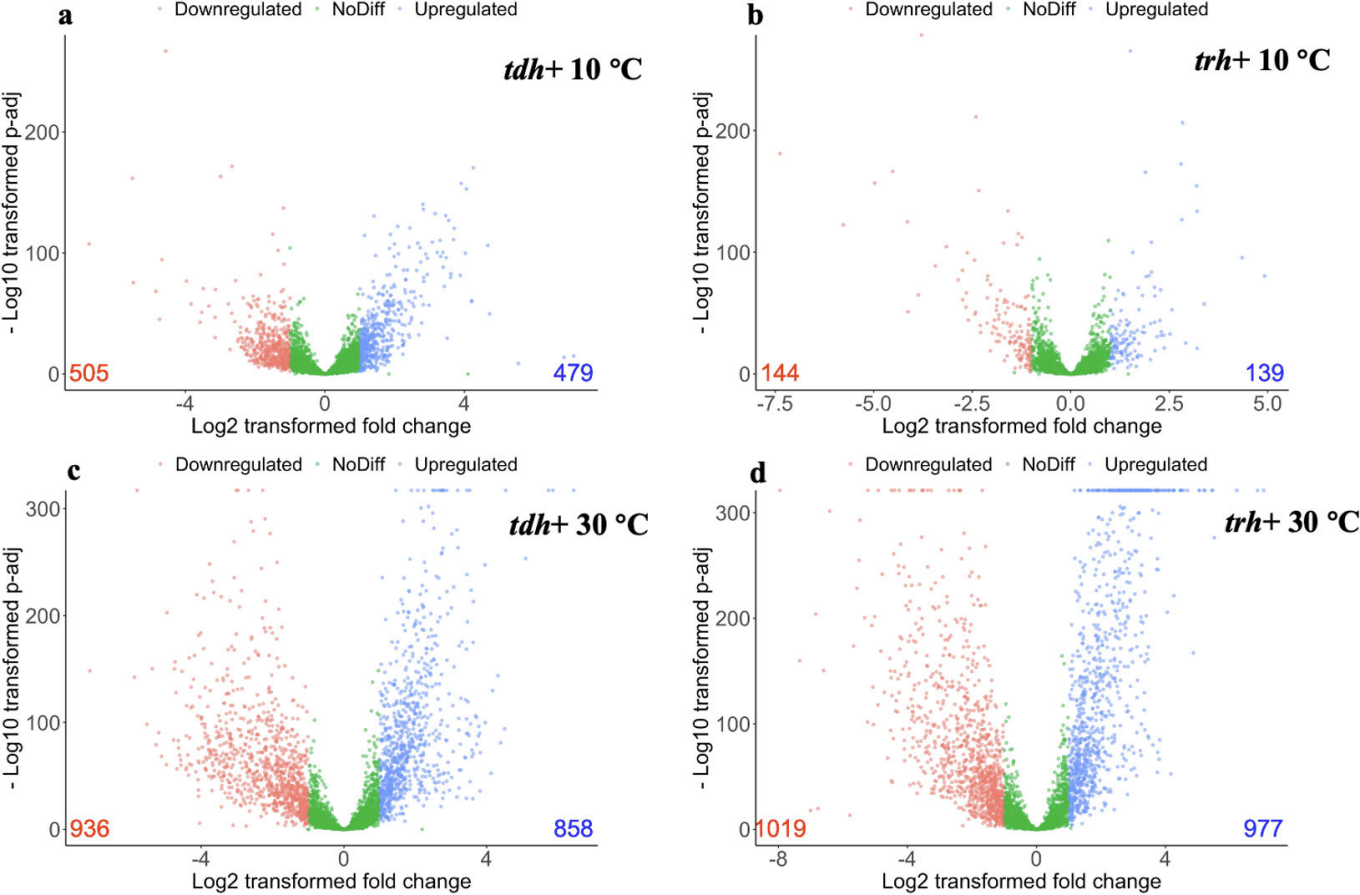
Differentially expressed genes of *V. parahaemolyticus* with *tdh*+ or *trh*+ genes when kept at 10°C (**a and b**) or 30°C (**c and d**) for 5 days in comparison with the control (*V. parahaemolyticus* kept in seawater for 2 hours). The x-axis represents the log_2_ of the fold change against the −log_10_ of the adjusted *P*-value. Red dots indicate the differentially expressed genes with at least a −1.0 change and statistical significance adjusted *P* < 0.05. Blue dots indicate the differentially expressed genes with at least a +1.0 change and statistical significance adjusted *P* < 0.05.

Differentially expressed genes (DEGs) at the transcriptomic level were further determined by RNA-seq analysis. Overall, more DEGs were detected in *Vp* transcriptome at 30°C compared with 10°C, which implies more active intracellular response at favorable temperature (Fig. 3). Venn graphs depicting unique and shared DEGs across different temperatures and strains were shown in Fig. S5. Notably, a substantial number of unique DEGs were identified when comparing the responses of the same strain at 10°C and 30°C. This disparity points toward distinct cellular reactions to differing temperature conditions. On the other hand, a significant overlap in DEGs was observed between *tdh*+ and *trh*+ strains at 30°C, indicating a similar pattern of gene regulation in response to optimal growth temperatures between two strains. In contrast, the shared DEGs between *tdh*+ and *trh*+ strains were fewer at 10°C. This divergence in shared DEGs across strains highlights the dissimilarity in the responses to low temperature. To validate the gene expression analyzed by RNA-seq, seven genes were randomly selected, and the gene expression was evaluated using quantitative real-time PCR (qRT-PCR). Results from qRT-PCR were consistent with that (upregulated or downregulated) of RNA-seq data analysis, suggesting the reliability of results from RNA-seq (Fig. S6).

### Cellular response of *Vp* adapting to 10°C seawater

**Fig 4:**
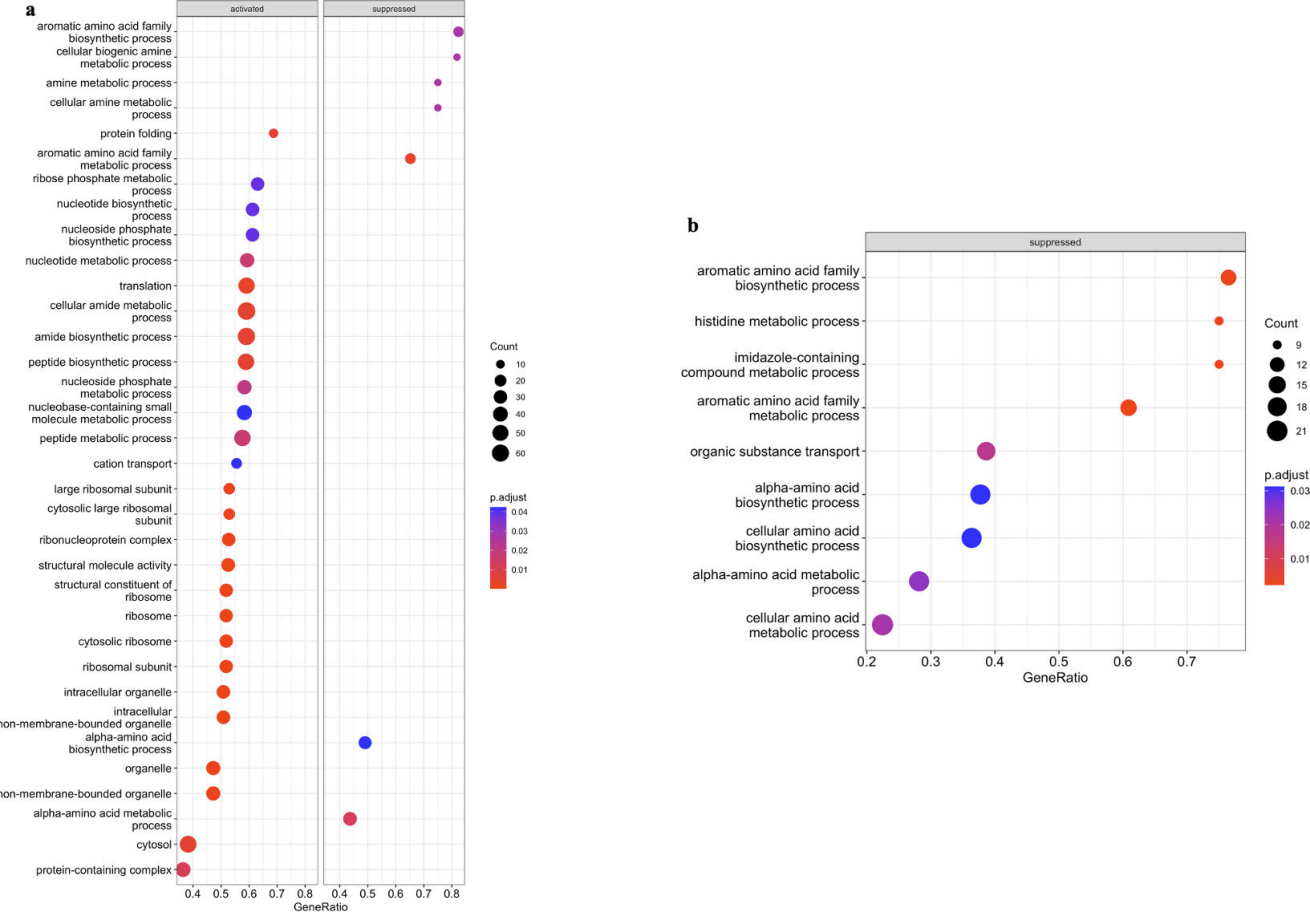
Gene set enrichment analysis against Gene Ontology database of *tdh+* (**a**) and *trh+* strains (**b**) when stored at 10°C in seawater for 5 days.

To decipher the biological processes, metabolism pathways, and virulence status in which the DEGs were implicated, gene set enrichment analysis (GSEA) was subsequently performed. Although more DEGs were observed in transcriptome of *trh*+ than *tdh*+ strain, fewer enriched Gene Ontology (GO) terms were enriched through GSEA in *trh*+ strain than *tdh*+ strain. Commonly observed in transcriptome profiles of both strains, the gene clusters associated with the biosynthesis of aromatic amino acid and alpha amino acid groups were significantly downregulated, indicating that *Vp* saved energy budget by avoiding expressing precursors of essential biomolecules (Fig. 4). Besides, functional gene clusters significantly enriched by aromatic amino acid biosynthesis, including those associated with organic substance transport and cellular amino acid biosynthetic process in *trh* strain, were all downregulated, which might suggest an inactive cellular status.

Previous studies have indicated that cellular strategies applied by *Vp* to address cold stress include upregulation of cold stress-related proteins and increase of membrane fluidity by enhancing fatty acid metabolism (17, 25, 26). Similar results were shown in this study. VP1889 encoding the cold shock protein A (*cspA*) was significantly upregulated for both strains (3.90 and 3.19 log_2_ fold change for *tdh*+ and *trh*+ strains, respectively) at 10°C (false discovery rate [FDR]-corrected *P*-values <0.05). CspA is an RNA chaperone that reduces RNA secondary folding caused by decreasing temperatures. The upregulation of *cspA* indicated that *Vp* counteracted the translational hardness caused by RNA folding at 10°C by increasing the *cspA* expression (27).

**Fig 5:**
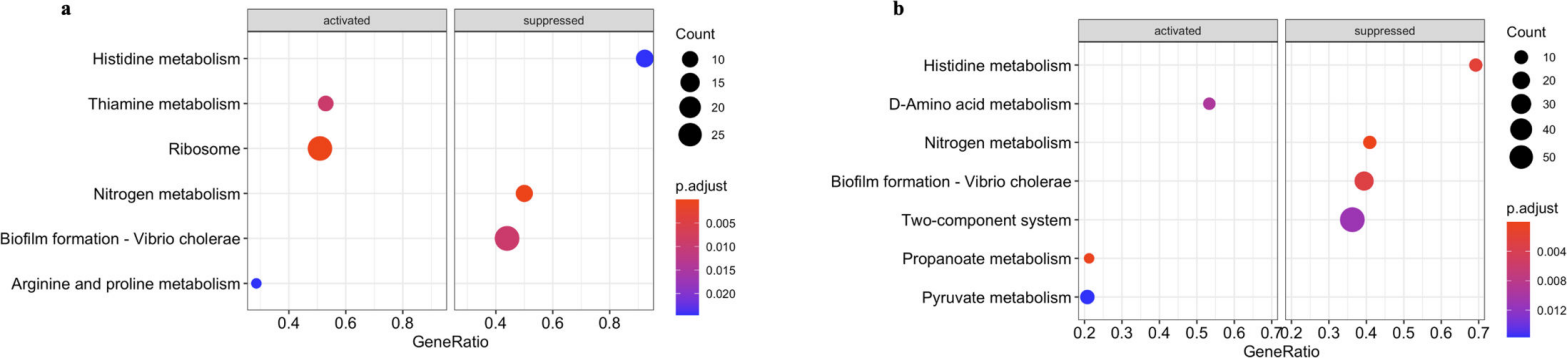
Gene set enrichment analysis against Kyoto Encyclopedia of Genes and Genomes database of *tdh+* (**a**) and *trh+* (**b**) strains when stored at 10°C in seawater for 5 days.

Modification of fatty acid is critical for bacterial survival at low temperatures, as lipid molecules can become more ordered and solidified as temperature decreases (28). Genes associated with the composition of cell membranes exhibited notable upregulation in both *tdh*+ and *trh*+ strains. These genes include but not limited to VPA 1151, which encodes a membrane-spanning protein, VP0767, responsible for encoding the outer membrane protein OmpA, as well as VPA0343, VP0818, and VPA0856, all of which encode potential membrane transporter proteins for *tdh*+ strain. For *trh*+ strain, VP0474 encoding probable membrane transporter protein and VPA0343 encoding putative membrane fusion protein were significantly upregulated. Xie et al. (17) pointed out that the essential role of co-occurred downregulated pyruvate metabolism and upregulated fatty acid biosynthesis in cold tolerance of *Vp* at 4°C. Although no direct evidence related to pyruvate metabolism and fatty acid biosynthesis was observed in transcriptome profile of *tdh*+ strain, thiamine metabolism was shown to be significantly upregulated (FDR-corrected *P*-values <0.05) (Fig. 5). Thiamine pyrophosphate is the key co-enzyme in fatty acid biosynthesis, which plays a fundamental role in generating energy (ATP), producing acetyl-CoA, and generating NADPH-critical components needed for the synthesis of lipids, including fatty acids (29). The observed upregulated thiamine metabolism pathway might suggest potential upregulated fatty acid biosynthesis in *tdh*+ strain in seawater at 10°C, and pyruvate metabolism was significantly upregulated in *trh*+ strain in seawater at 10°C (Fig. 5). At the proteomic level, Tang et al. (30) reported that pyruvate dehydrogenase complex (PDHC) repressor (a regulator negatively impacts the formation of PDHC) was mostly downregulated in *Vp* incubated at 4°C after 18 hours and suggested that the resulted enhanced PDHC activity was critical for *Vp* to maintain its viability under cold stresses. Taken together, these results highlighted the active pyruvate metabolism change involved in *Vp* surviving at low temperatures.

### Cellular responses of *Vp* growing at 30 °C

**Fig 6:**
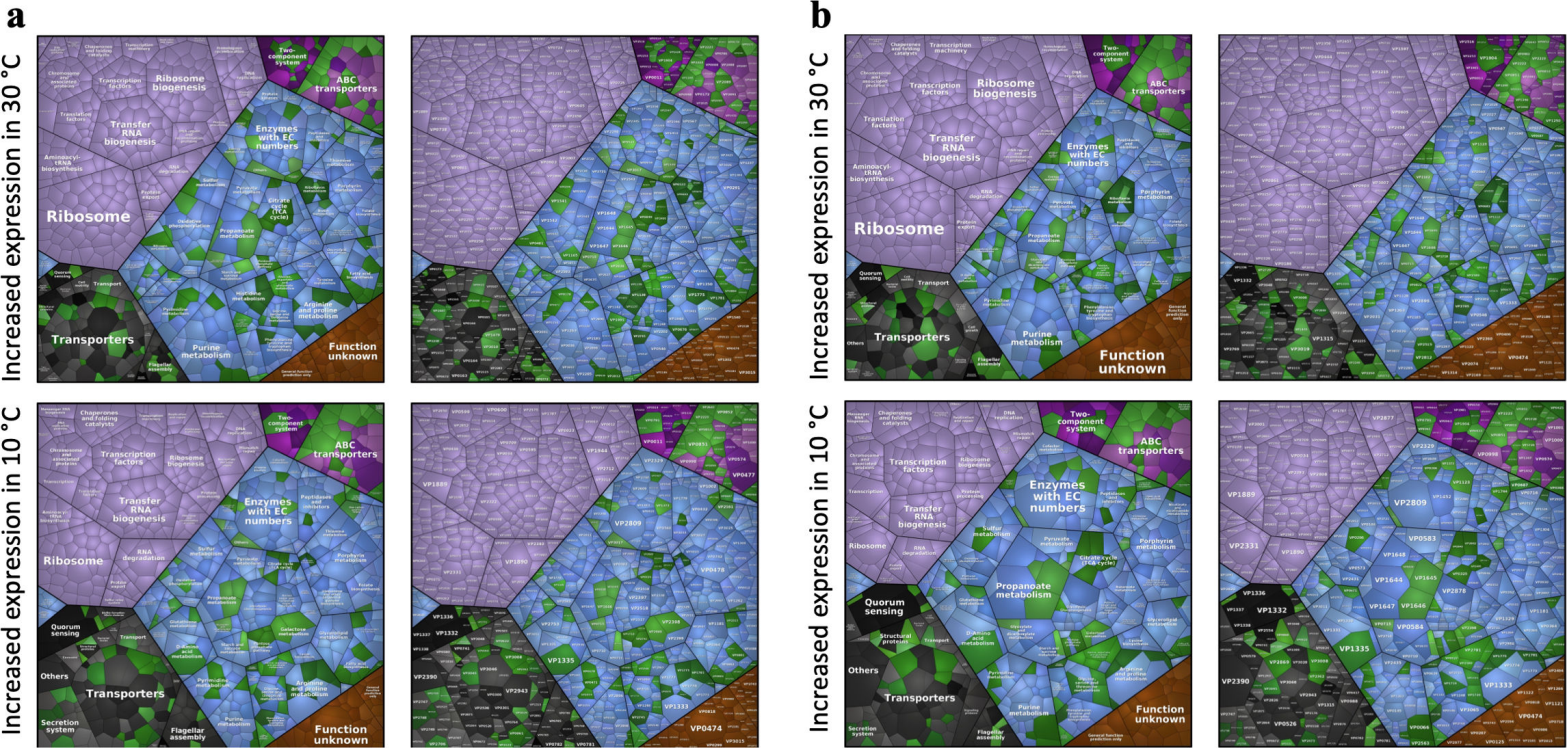
Proteomap illustrating differentially expressed genes of *tdh*+ strain (a) and *trh*+ strain (b) when stored at 10°C or 30°C for 5 days. Genes are clustered by different functional groups.

**Fig 7:**
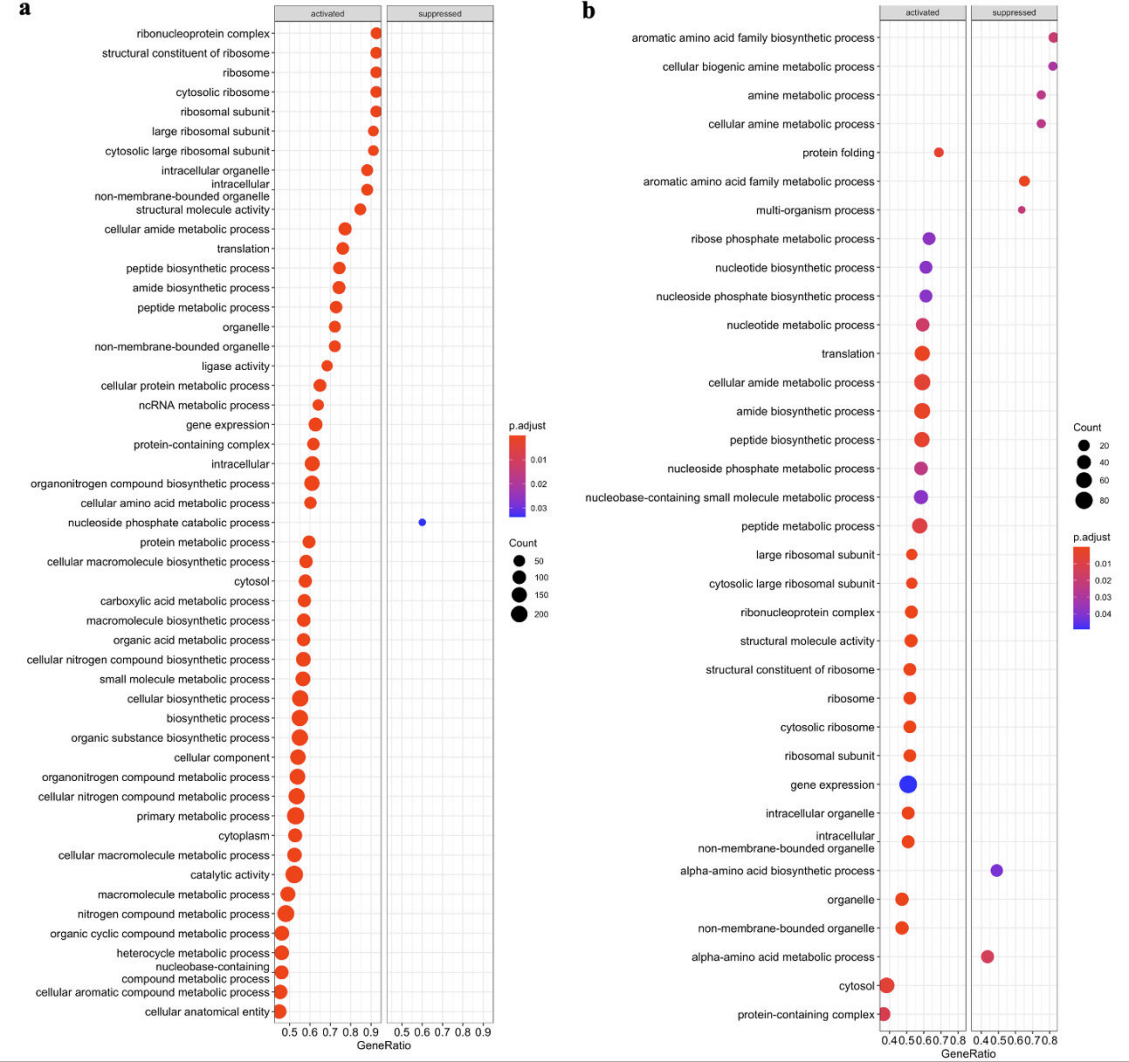
Gene set enrichment analysis against GO database of *tdh+* (**a**) and *trh+* (**b**) strains when stored at 30°C in seawater for 5 days.

**Fig 8:**
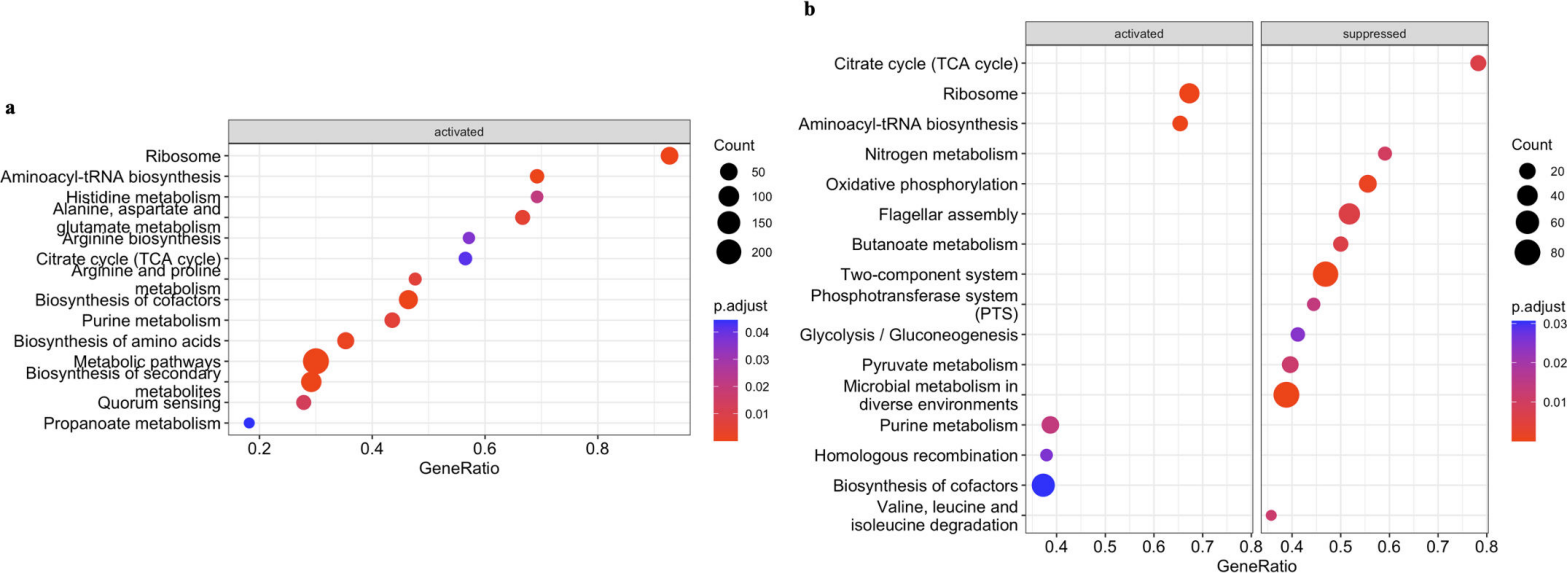
Gene set enrichment analysis against KEGG database of *tdh+* (a) and *trh+* (b) strains when stored at 30°C in seawater for 5 days.

A strong signature indicating *Vp* adapting to preferable environment was evident, including ribosome biogenesis, amino acid metabolism, and purine metabolism (Fig. 6). For both *tdh*+ and *trh*+ strains, genes annotated to ribonucleoprotein complex and large ribosomal subunit were significantly enriched based on GSEA-GO results (Fig. 7). Significant activation of cofactor biosynthesis pathway was observed in both *tdh+* and *trh*+ strains based on GSEA-Kyoto Encyclopedia of Genes and Genomes (KEGG) results (Fig. 8). These findings could imply elevated ribosomal activity to support cellular growth and division during the exponential phase. Greater than 50% of DEGs were annotated to biosynthesis processes, including the macromolecule biosynthetic process, cellular biosynthetic process, and organic substance biosynthetic process that were significantly upregulated in *tdh*+ strain (Fig. 7). Amino acid biosynthesis pathway and alanine, aspartate, and glutamate metabolism pathways were significantly upregulated (Fig. 8). Alanine, aspartate, and glutamate are critical amino acids serving as precursor for diverse metabolites as essential cellular component for bacterial cell growth (31, 32). In addition, both arginine biosynthesis and arginine metabolism were both significantly upregulated (Fig. 8). Similar results were reported in the previous work: Li et al. (33) reported that arginine biosynthesis pathway was upregulated in *Vp* incubated in eutrophic outlet water at 30°C compared with counterparts incubated at 16°C. Arginine biosynthesis is essential to microbial growth as arginine can be converted to putrescine, which serves as an essential regulator for cell growth, differentiation, proliferation, and various physiological processes (34, 35). When microbial growth shifts from exponential phase to stationary phase, the expression of growth-associated genes is downregulated; meanwhile, persistence-associated genes are upregulated so that bacterial cells can remain metabolically active in stationary phase (36). These upregulated biosynthetic processes contributed to the fitness of *tdh*+ strain at optimum growth temperature and maintaining stable populational level at the stationary phase.

Higher growth rate was observed in *tdh*+ strain in comparison to *trh+* strain at the phenotypic level. Results at transcriptomic levels provided additional biological insights. More significantly downregulated functional gene clusters and metabolism pathways were detected in *trh+* strain than *tdh*+ strain based on GSEA results (Fig. 7 and 8). Functional gene clusters associated with ribosome, ribosome biosynthesis, and transfer RNA biosynthesis coupled with pathway enrichment in central energy metabolism reflect energy use for cell growth and proliferation of *tdh*+ strain at 30°C. Central energy metabolisms including glycolysis/gluconeogenesis, pyruvate metabolism, and TCA cycle were significantly downregulated in the *trh+* strain after 5 days of incubation at 30°C, suggesting that the energy generation was weakened in *trh+* strain. Moreover, oxidative phosphorylation pathway was significantly downregulated in *trh+* strain as well, indicating that the process of intracellular ATP synthesis was inhibited. In addition to downregulation of energy metabolism pathways, significantly downregulated usage of valine, leucine, and isoleucine pathway was detected in *trh+* strain (Fig. 8). Valine, leucine, and isoleucine are the core branched-chain amino acids essential for bacterial growth (37). Such decreased degradation of branched-chain amino acid might inhibit interconversion to metabolites essential for growth and co-factors. This observed *trh+* strain-only inferiority due to turned off central energy metabolism might explain its lower fitness in comparison to *tdh*+ strain in seawater at 30°C.

### Expression of virulence genes at different temperatures

Microbial pathogenesis can be significantly affected by environmental temperature (38). Virulence and pathogenesis of *Vp* were commonly reported based on studies using live animal models (39 – 41). However, limited information about its virulence in natural seawater environment has been reported at this moment. Based on the GSEA-KEGG results, the biofilm formation pathway was significantly downregulated at 10°C; this has been observed in both *tdh*+ and *trh*+ strains. Han et al. (42) reported that the biofilm formation of *Vp* on food and food surfaces increased as the environmental temperatures increased. Both results highlight the importance of temperature during both pre-harvest production and handling and post-harvest processing and storage. Moreover, histidine metabolism was significantly downregulated in both strain *Vp* at 10°C (Fig. 5). It is essential to explore the impact on downstream metabolites in the histidine metabolism pathway, including compounds like histamine, a significant allergen found in aquaculture products (43). For a more comprehensive understanding, metabolomic profiling of *Vp* in seawater at low temperature warrants additional future studies. This approach would provide detailed valuable insights into the broader metabolic changes occurring in response to environmental variations (44, 45).

Urmersbach et al. (25) reported that expression of major virulence-associated genes of *Vp* RIMD 2210633 such as *tdh*, *tox*R, and *tox*S remained unaffected by cold and heat shock in alkaline peptone water (4°C and 42°C, respectively). In this study, our results showed that virulence-associated genes, including *toxR*, *toxS*, and T3SS1 effectors *vopQ*, *vopR*, *vopS*, and VPA0450, showed less than 1.0 log_2_ fold change. The RNA-seq analysis did not reveal the expression of VPA1378 (a *tdh*1 gene harbored by ATCC 43996 *tdh*+ strain) or expression of VPA1314 (a *tdh*2 gene harbored by ATCC 43996 *tdh*+ and ATCC 17802 *trh*+ strains). The undetected gene expression could potentially result from the absence of a suitable host, and *Vp* might prioritize energy allocation to ensure long-term persistence in nutrient-limited seawater (46). Less expression of virulence-associated genes was expected when bacteria need to actively regulate genes coding for enzymes essential for growth and persistence in the optimal environment (47).

Moreover, VP1890 (*vacB*) encoding a putative virulence-associated protein was significantly upregulated for *tdh*+ and *trh*+ strains at both 10°C and 30°C (with 4.25 and 2.13 log_2_ fold changes for *tdh*+ and *trh*+ strains at 10°C, respectively; 4.27 and 3.42 log_2_ fold change for *tdh*+ and *trh*+ strains at 30°C, respectively, FDR-corrected *P*-values <0.05). The product of *vacB* was reported to be an exoribonuclease RNase contributing to the virulence of *Shigella* and enteroinvasive *Escherichia coli* (48). The strong expression of *vacB* in *Vp* was consistent with previous studies. Meng et al. (49) reported more than 5 log_2_ fold change upregulation of *vacB* in viable but non-culturable state *Vp* induced at 4°C over 40 days. Urmersbach et al. (25) also reported that *vacB* showed the highest upregulation (7.01 log_2_ fold change) in *Vp* when being incubated in alkaline peptone water at 15°C for 30 min. All combined information confirmed high expression levels of *vacB* at temperatures ranging from 4°C to 15°C. In this study, the expression of *vacB* was also upregulated at 30°C. As discussed by Liao et al., the detection of *Vp* at lower temperatures can be challenging; newer candidate genes that continuously expressed at higher levels at various temperatures are needed. Through this study, results indicated that *vacB* could serve as a potential biomarker to identify *Vp* in natural coastal environment across seasons. Through BLAST, the *vacB* nucleotide sequence shows high specificity in *Vp* (Table S1).

### Conclusion

This study investigated the fitness at the phenotypic level and the cellular response at the transcriptomic level of two *Vp* strains (ATCC 43996 *tdh*+ and ATCC 17802 *trh*+) when kept in natural seawater of different temperatures (10°C and 30°C). At the phenotypic level, both *tdh*+ and *trh*+ strains persisted for over 10 days when kept at 10°C. The survival model indicated a higher die-off rate for the *trh*+ strain compared to the *tdh*+ strain. When kept at 30°C, the growth of *tdh*+ strain was better than the *trh*+ strain by showing a higher growth rate. Based on the transcriptomic analysis, more DEGs were detected at 30°C than 10°C, indicating that cellular responses of *Vp* were more complex during summer months. At 10°C, while no remarkable expression of virulence-associated genes was observed, the expression of cold shock-associated genes was upregulated. In addition, both strains showed downregulated biofilm formation pathway and histidine metabolism at 10°C. The expression of genes encoding major hemolysin was not detected in *tdh*+ and *trh*+ strains at both temperatures, highlighting the cost-effective energy allocation strategy by *Vp* during growth and persistence in seawater. The *vac*B gene encoding a putative virulence-associated protein (VP1890) presented significantly upregulated expression in *tdh*+ and *trh*+ strains at both 10°C and 30°C; the identification of this gene warrants further analysis to see if it can be used as a new alternative target for the detection of pathogenic *Vp*. In summary, this study generated crucial and valuable information about the fitness of *Vp* in natural pre-harvest environment. Information will directly support *Vp* risk assessment, management, and detection.

## MATERIALS AND METHODS

### Culture preparation and incubation conditions

Frozen cultures of *Vp* strains ATCC 43996 (*tdh*+, Cockles causing fatal food poisoning) and ATCC 17802 (*trh*+, Shirasu food poisoning) purchased from ATCC were streaked and activated on tryptic soy agar (TSA) supplemented with 3% NaCl. Cultures were incubated at 37°C for overnight. After that, a single colony was picked from each TSA plate and transferred into 10 mL of tryptic soy broth (TSB) supplemented with 3% NaCl for additional 24 hours of incubation at 37°C. After incubation, a loopful of fresh liquid culture was transferred into another 10 mL of fresh TSB supplemented with 3% NaCl. The inoculated TSB was incubated at 37°C for overnight and washed the next day by centrifugation (Eppendorf, Hauppauge, NY, USA) at 3,000 × *g* for 10 min. Washed cultures were resuspended with 2 mL of phosphate-buffered saline (PBS; pH 7.4). The optical density at 600 nM (OD600) of each washed culture was adjusted to 1.6 ± 0.1 by using a spectrophotometer (Thermo Scientific, Piscataway, NJ, USA). This washed and adjusted culture had ca. 7.0 log CFU/mL of cells based on plate count results. To inoculate the seawater, 1 mL of washed culture was added into 9 mL of autoclaved natural seawater. Seawater was collected from the from the Auburn University Marine Extension and Research Center located in Dauphin Island, AL. The pH and salinity levels of collected seawater were measured using pH and salinity meter (YSI Pro1030 pH or ORP, Conductivity, Salinity Instrument, Washington, DC, USA) according to manufacturer’s instruction. Inoculated seawater samples were first kept at ambient temperature for 2 hours to enable the *Vp* cultures to adapt to the new environment. After 2 hours, inoculated seawater samples were stored at 10°C for 10 days or 30°C for 5 days without agitation.

### Enumeration of *Vp* in inoculated seawater samples by using the plate count method

During storage, sub samples (1 mL each) were taken and plated every 24 hours for the 10°C storage condition and were plated every 2 hours for the first 12 hours then at hours 24, 48, 72, and 120 for the 30°C storage condition. Three biological replicates were conducted. Every 1 mL of seawater sample was diluted in serial 10-fold dilutions and plated onto thiosulfate-citrate-bile salts-sucrose plates (BD, Sparks, MD, USA). Plates were incubated at 37°C for 18 hours before enumeration. Plates were then placed back to the incubator, and the colony counts were confirmed after another 24 hours of incubation. *Vp* concentrations were expressed in common logarithm transformation format with the unit of CFU/mL.

### Statistical analysis and survival and growth models for describing *Vp* behaviors in seawater

The populations of *Vp* present in seawater were enumerated at different time intervals at 10°C and 30°C. One-way analysis of variance followed by the Tukey test was applied to compare the difference in *Vp* concentrations as predicted by the survival and growth models. The models were established for two *Vp* strains at two storage temperatures with the OriginPro 2023 software (OriginLab Corporation, Northampton, MA, USA). *P* < 0.05 was considered statistically significant. The Baranyi model (see equation 1) was chosen to fit the *Vp* population data, and the calculations were performed using the DMfit tool available at the Combase website, https://browser.combase.cc/ (50). The equation of the Baranyi model is as follows:


$$y\left(t\right)=y_{0}+\;\mu_{min}A\left(t\right)-\;\frac{1}{m}ln\left(1+\frac{e^{m\mu }{min}^{A\left(t\right)}-1}{e^{m}\left(y_{end}-y_{0}\right)}\right)$$

$$A\left(t\right)=t+\frac{1}{v}\ln\left(\frac{e^{-vt}+q_{0}}{1+q_{0}}\right)$$

where y is the natural logarithm of the bacteria concentration at any given time (ln CFU per milliliter), $y_{0}$ and $y_{end}$ are the initial value and the end value of y, $A\left(t\right)$ is the equation governing the duration of the period preceding the log linear inactivation phase, t is time (day), m determines the smoothness of the transition from the exponential inactivation phase to the survival tail, $\mu_{min}$ is the minimum value of the inactivation rate or the maximum value of the growth rate, v is the rate at which the bacteria lose the ability to survive during the shoulder, and $q_{0}$ is the initial physiological state of bacterial cells.

### RNA extraction and sequencing

RNA samples extracted from *Vp* 2 hours after inoculation were labeled as the control, and RNA samples extracted from *Vp* strain at the end of 5 days of storage at 10°C or 30°C were labeled as test group. Five days were chosen as it was expected that *Vp* should already have been in a stasis stage during that time (25). To extract the RNA, 1 mL of *Vp* culture was taken and centrifuged at 3,000 × g for 10 min. The supernatant was removed, and the cell pellet was re-suspended in 1 mL of PBS. The total bacterial RNA was extracted by using the Qiagen RNeasy mini kit (Qiagen, Valencia, CA) following the manufacturer’s instruction. The quality of the extracted RNA was measured with the Agilent 2100 electrophoresis bioanalyzer (Agilent, Santa Clara, CA) to ensure that the RNA integrity numbers of all RNA samples were greater than 7.0. Once the RNA quality was confirmed, cDNA library was prepared by using the QuantiTect reverse transcription kit (Qiagen, Valencia, CA). The cDNA library was then sent to the Genomic Services Laboratory at HudsonAlpha Genome Sequencing Center (Huntsville, AL) and sequenced on the Illumina HiSeq 2500 platform to generate 150-bp pair-end reads.

### Transcriptomic analysis

A schematic illustration of transcriptomics analysis pipeline is shown in Fig. S1. The quality of raw reads was checked by FASTQC. Given the small size of bacterial genome (approximately 5.1 Mb for *Vp*) and since there was no potential mRNA splicing issues, a fast and bias-aware analytical pipeline, Salmon, was used to achieve quantification of transcript expression mapping (51, 52). RIMD 2210633 was selected as the reference strain because of its established status as a reference in NCBI and its use in the subsequent KEGG analysis. The genome similarity among ATCC 17802, ATCC 43996, and RIMD 2210633 was calculated by using sourmash (k = 31, --estimate-ani) (53). Salmon was used to align the reads against the *Vp* RIMD 2210633 protein coding sequence (CDS) region of each gene (reference transcriptome [GenBank accession number GCA_000196095.1]) with parameters -gcBias. Read counts were imported into R, filtered, and mapped mRNA transcripts to gene ID by using the tximport package (54). Normalized gene counts were calculated to account for differentially expressed genes among samples using output matrix from Deseq 2 (55). Genes with an FDR-corrected *P*-values <0.05 were considered significant. Threshold of log_2_ fold change ≥1 was considered upregulated, and threshold of log_2_ fold change ≤1 was considered downregulated (17). To gain insights into the intrinsic patterns and relationships within the gene expression profiles, a PCA was performed on the regularized log-transformed genes commonly shared among *tdh*+, *trh*+, and RIMD 2210633 strains. Reference transcriptome annotation against Gene Ontology database was conducted by using eggnog-mapper (56). GSEA was performed for genes that were upregulated and downregulated in both storage temperatures against the Gene Ontology (ont = ALL) and the Kyoto Encyclopedia of Genes and Genomes (organism = vpa) databases by using the R package clusterProfiler (57). Differentially expressed genes were visualized by using proteomaps (58). The reference gene list of *Vp* used in proteomaps was constructed based on the JSON file in the KEGG orthology database by Python (treemap template ID: *Vibrio parahaemolyticus* RIMD 2210633 V7).

### qRT-PCR validation

To validate the transcriptomic results, genes of each *Vp* strain showing the same upregulation or downregulation patterns at both 10°C and 30°C were selected for validation studies. The gene *pvuA* was used as the housekeeping reference gene as its expression level has shown to be stable through a wide range of temperatures (59). The expression level of the select gene was normalized to the reference gene and calculated by using the −ΔΔCt method (60). The list of primers used in this study is provided in Table 2. qRT-PCR was conducted on the QuanStudioTM Real-Time PCR system (Applied Biosystems, Foster City, CA). The total volume of each reaction was set to 25 µL, consisting of 2-µL cDNA aliquot (concentration 1 ng/µL), 1 µL forward primer (1 µM), 1-µL reverse primer (1 µM), 12.5-µL 2× SYBR Green PCR Master Mix (Life Technologies, Carlsbad, CA, USA), and 8.5-µL nuclease-free water. Program of thermocycler was set to start with an initial denaturing period at 95°C for 10 min and then 40 cycles at 95°C for 15 s, 52°C for 20 s, and 72°C for 25 s. The specificity of the PCR product was checked by analyzing the melt curve.

**TABLE 2:** Primers used in qRT-PCR analysis for the validation of randomly selected differentially expressed genes identified by RNA-seq

Gene	ID	Encoding protein	Sequence 5′ to 3′	Size (bp)	Reference	Condition
pvuA	VPA1656	Ferric vibrioferrin receptor	CAAACTCACTCAGACTCCACGAACCGATTCAACACG	156	(61)	All
	VP1332	Binding protein component of ABC transporter	ATCGTCGTATCGACCGTCTTAGCTAGTAGGCGGTAAACTTCGTCAG	193	This study	trh+_10&30
ocd2	VP1333	Ornithine cyclodeaminase Ocd2	GTACTGGCAACTTAGCCCCTTAAGACACAGAGAACTGTCGCTCTTC	170	This study	trh+_10&30
acnD	VP1646	Aconitate hydratase	GTACCGGAAGAGGACTTCAACTCTCCACATACGTACAACCTGACCTTC	174	This study	trh+_10&30
dnaA	VP0011	Chromosomal replication initiator protein	GCTTCAAGAAGAGCTACCAGCTACGGCGCGAATAGAGTGAGAGTAT	93	This study	trh+_10&30
	VP0474	Probable membrane transporter protein	GGTGGAGTTGGTTTCTACGATGCCATACAGGTAACCCTGCTAGAAC	180	This study	tdh+_10&30
	VPA1121	Putative acyl-CoA dehydrogenase	GGTGGCTATGGCTACATCAAAGGCTCTACGTCTTCCGTGAGTAAAC	136	This study	tdh+_10&30
	VPA1060	Putative two-component response regulatory proteins	GCTCTTCAACCTTGGATTGACCTGTACGCGTGTTCCTCATCTAC	166	This study	trh+_10&30tdh+_10&30
glgC	VPA0833	Glucose-1-phosphate adenylyltransferase	GAAAACCCACCTACTCTTCCAGACGTCATGGCTAGACGTTTCCAGT	129	This study	trh+_10&30tdh+_10&30

## Data Availability

The raw and processed RNA-seq data can be found in the GEO database under accession numbers PRJNA949728 and PRJNA949727.

## SUPPLEMENTAL MATERIAL

Supplemental figures spectrum.02783-23-s0001.pdf Figures S1 to S6.

Supplemental table spectrum.02783-23-s0002.docx Table S1

ASM does not own the copyrights to Supplemental Material that may be linked to, or accessed through, an article. The authors have granted ASM a non-exclusive, world-wide license to publish the Supplemental Material files. Please contact the corresponding author directly for reuse.
