# Supplementary material for: Fitness and transcriptomic analysis of pathogenic Vibrio parahaemolyticus in seawater at different shellfish harvesting temperatures
Source: Microbiol Spectr. 2023 Nov 14;11(6):e02783-23. doi: 10.1128/spectrum.02783-23 (PMC10715093; doi:10.1128/spectrum.02783-23)
Supplement: Supplemental figures — Figures S1 to S6. [file spectrum.02783-23-s0001.pdf]

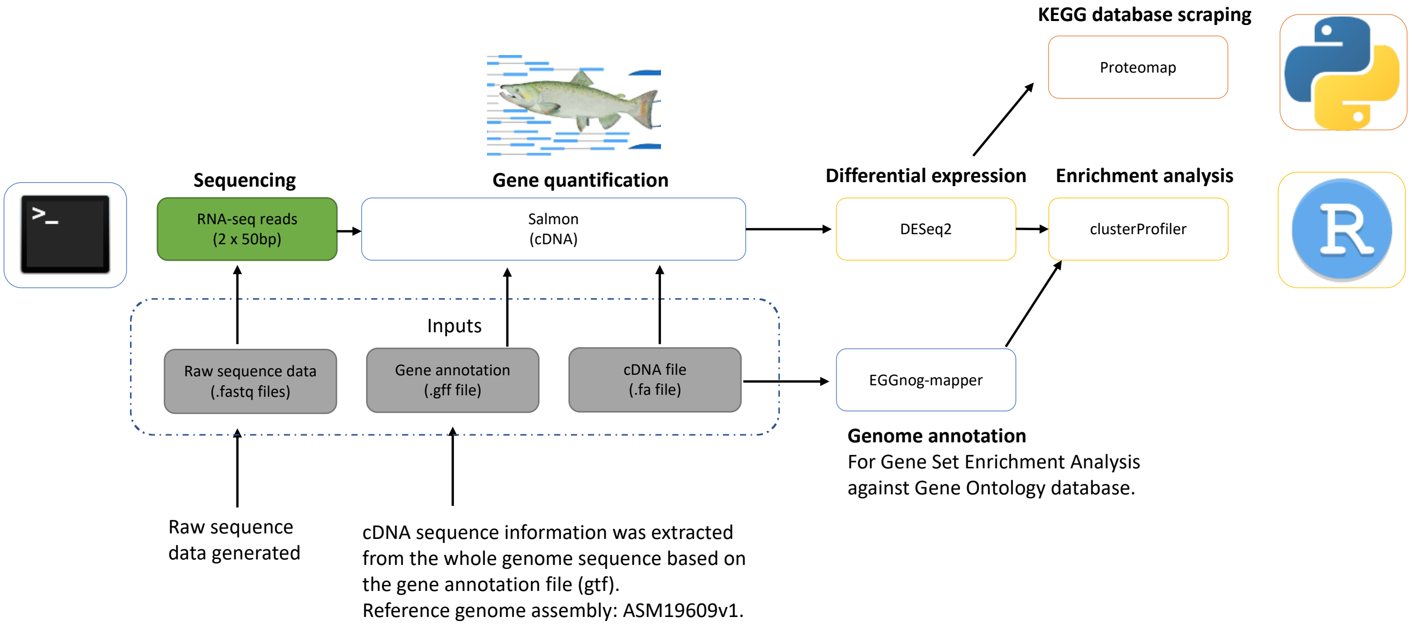

Figure S1. A schematic illustration of the analytical pipeline for the transcriptomic data. Blue color indicates steps conducted in linux system. Yellow color indicates steps conducted in R. Orange color indicates steps conducted in Python.

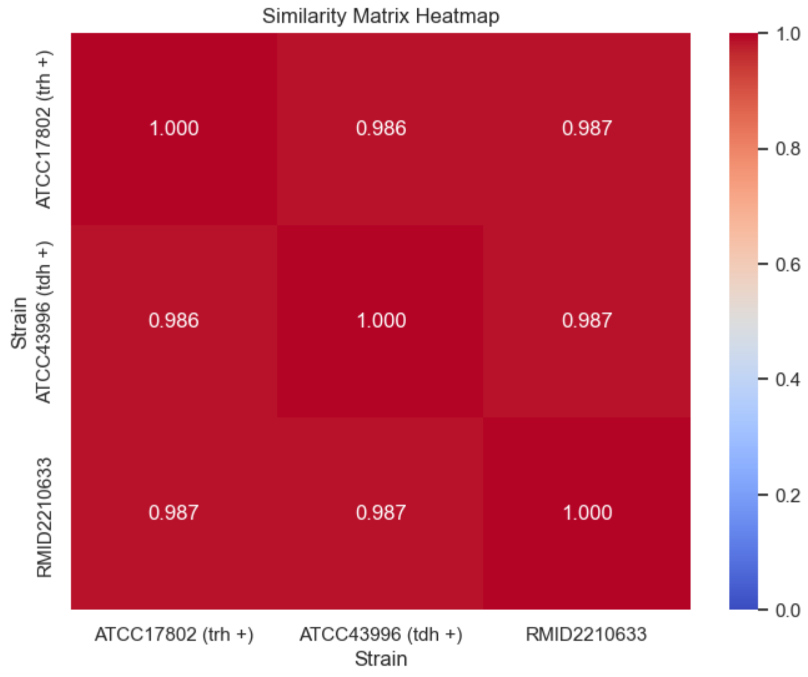

Figure S2. Genome similarity among *tdh*+, *trh*+ strians, and RMID 2210633 strains was analyzed by using the sourmash software with k being 31 based on the average nucleotide identity.

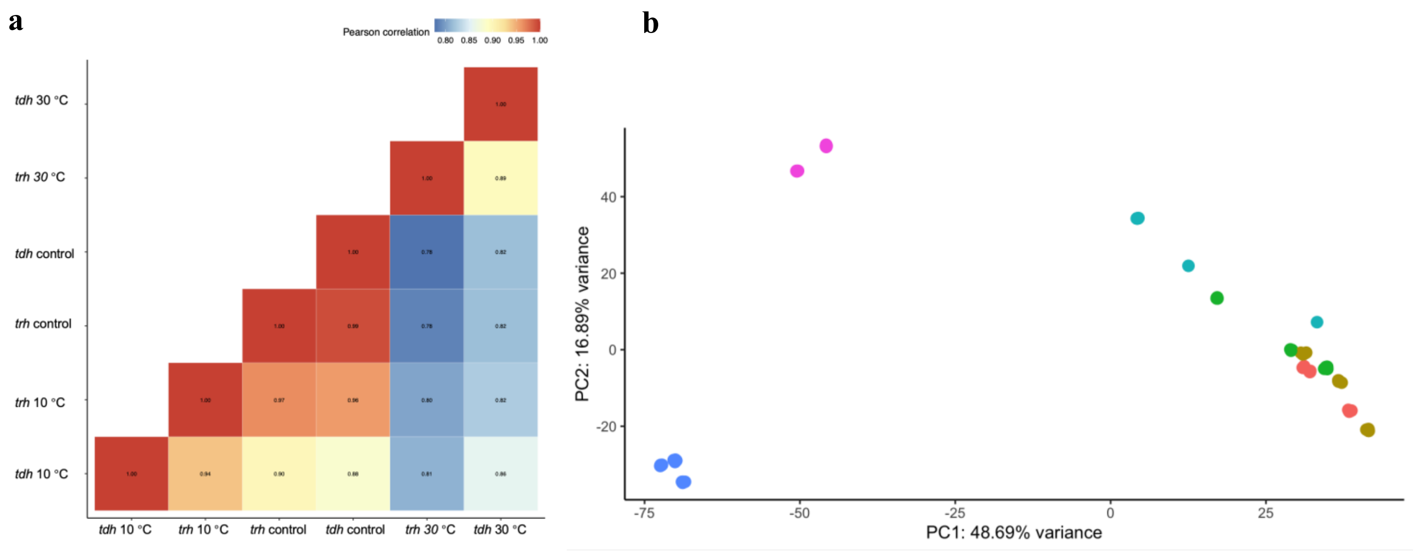

Figure S3. Pearson correlation (a) of transcriptomic profiles *V. parahaemolyticus* in the control group, 2 hours after seawater inoculation (*tdh*+ control and *trh*+ control), at 10 °C (*tdh*+ 10 °C and *trh*+ 10 °C after five days incubation), and at 30 °C (*tdh*+ 30 °C and *trh*+ 30 °C after five days incubation*)*; Principal component analysis (b) of transcriptomic profiles *V. parahaemolyticus* in the control group, 2 hours after seawater inoculation (*tdh*+ and *trh*+), at 10 °C (*tdh*+ and *trh*+), and at 30 °C after five days incubation (*tdh*+ and *trh+)*

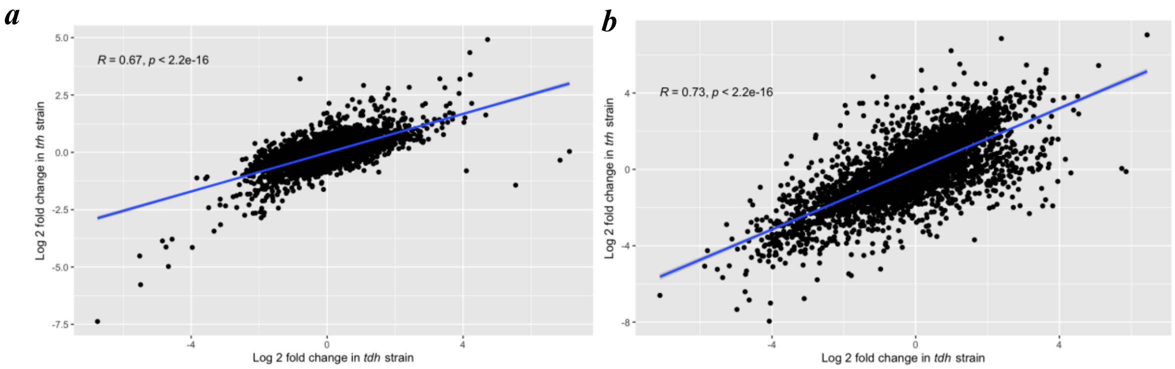

Figure S4. Scatter plots of each gene expression levels between the *tdh*+ and *trh*+ strains at 10 °C after five days incubation (a) and 30 °C after five days incubation (b).

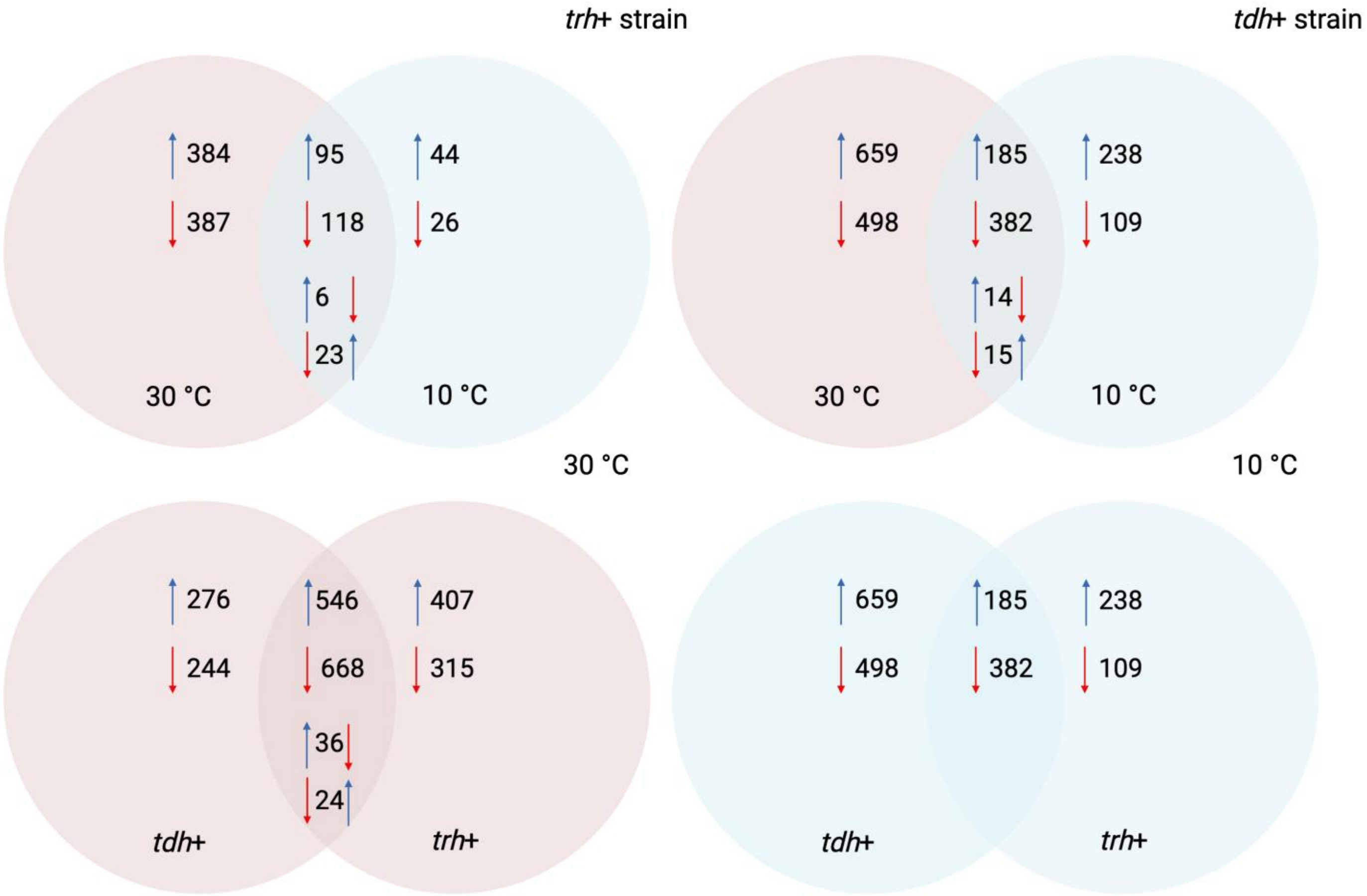

Figure S5. Venn diagrams depicting Differentially Expressed Genes across strains and temperatures. Created with BioRender.com

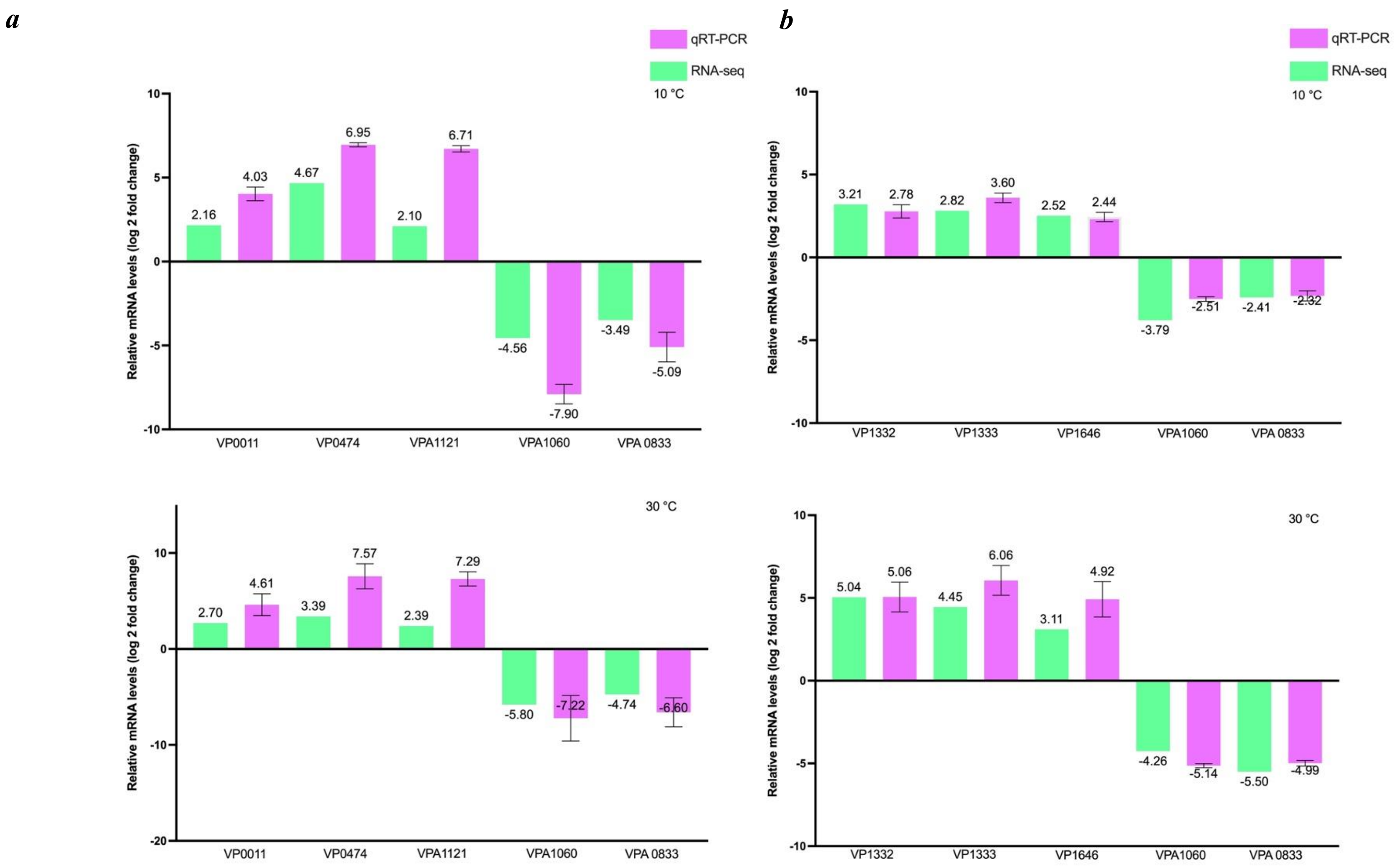

Figure S6 Validation of the select differentially expressed genes identified from RNA-seq results by qRT-PCR for *tdh*+ strain (a) and *trh*+ strain (b).
