# Supplementary material for: Fitness and transcriptomic analysis of pathogenic Vibrio parahaemolyticus in seawater at different shellfish harvesting temperatures
Source: Microbiol Spectr. 2023 Nov 14;11(6):e02783-23. doi: 10.1128/spectrum.02783-23 (PMC10715093; doi:10.1128/spectrum.02783-23)
Supplement: Supplemental table — Table S1 [file spectrum.02783-23-s0002.docx]

**Table S1.** Comparative Analysis of *vacB* Gene Sequences using Blast Alignment

| Description | Scientific Name | Max Score | Total Score | Query Cover | E value | Per. ident | Acc. Len | Accession |
| --- | --- | --- | --- | --- | --- | --- | --- | --- |
| *Vibrio parahaemolyticus* strain VPD14 chromosome 1, complete sequence | *Vibrio parahaemolyticus* | 4111 | 4111 | 100% | 0 | 100 | 3291121 | CP031781.1 |
| *Vibrio parahaemolyticus* strain FDAARGOS_191 chromosome 1, complete sequence | *Vibrio parahaemolyticus* | 4111 | 4111 | 100% | 0 | 100 | 3295711 | CP020427.2 |
| *Vibrio parahaemolyticus* strain HZ chromosome 1, complete sequence | *Vibrio parahaemolyticus* | 4111 | 4111 | 100% | 0 | 100 | 3305216 | CP130651.1 |
| *Vibrio parahaemolyticus* strain JX-FB-vp35 chromosome 1 | *Vibrio parahaemolyticus* | 4111 | 4111 | 100% | 0 | 100 | 3288658 | CP093274.1 |
| *Vibrio parahaemolyticus* strain RMDVP1 chromosome 1, complete sequence | *Vibrio parahaemolyticus* | 4111 | 4111 | 100% | 0 | 100 | 3300152 | CP102434.1 |
| *Vibrio parahaemolyticus* strain VP20210406 chromosome 1 | *Vibrio parahaemolyticus* | 4111 | 4111 | 100% | 0 | 100 | 3288558 | CP107279.1 |
| *Vibrio parahaemolyticus* RIMD 2210633 DNA, chromosome 1, complete sequence | *Vibrio parahaemolyticus* RIMD 2210633 | 4111 | 4111 | 100% | 0 | 100 | 3288558 | BA000031.2 |
| *Vibrio parahaemolyticus* strain LVP66 chromosome 1, complete sequence | *Vibrio parahaemolyticus* | 4095 | 4095 | 100% | 0 | 99.87 | 3325904 | CP040103.1 |
| *Vibrio parahaemolyticus* strain 2013V-1174 chromosome 1, complete sequence | *Vibrio parahaemolyticus* | 4084 | 4084 | 100% | 0 | 99.78 | 3369685 | CP046787.1 |
| *Vibrio parahaemolyticus* strain FORC_006 chromosome 1, complete sequence | *Vibrio parahaemolyticus* | 4084 | 4084 | 100% | 0 | 99.78 | 3376731 | CP009765.1 |
| *Vibrio parahaemolyticus* O1:K33 str. CDC_K4557 chromosome I, complete sequence | *Vibrio parahaemolyticus* O1:K33 str. CDC_K4557 | 4084 | 4084 | 100% | 0 | 99.78 | 3331580 | CP006008.1 |
| *Vibrio parahaemolyticus* strain AM51552 chromosome 1, complete sequence | *Vibrio parahaemolyticus* | 4061 | 4061 | 100% | 0 | 99.6 | 3293962 | CP046760.1 |
| *Vibrio parahaemolyticus* strain SHP/2 chromosome 1 | *Vibrio parahaemolyticus* | 4061 | 4061 | 100% | 0 | 99.6 | 3247960 | CP066156.1 |
| *Vibrio parahaemolyticus* strain 2012AW-0224 chromosome 1, complete sequence | *Vibrio parahaemolyticus* | 4050 | 4050 | 100% | 0 | 99.51 | 3535345 | CP046831.1 |
| *Vibrio parahaemolyticus* strain AM46865 chromosome 1, complete sequence | *Vibrio parahaemolyticus* | 4050 | 4050 | 100% | 0 | 99.51 | 3284504 | CP046761.1 |
| *Vibrio parahaemolyticus* strain LVP1 chromosome 1, complete sequence | *Vibrio parahaemolyticus* | 4050 | 4050 | 100% | 0 | 99.51 | 3478693 | CP040100.1 |
| *Vibrio parahaemolyticus* strain 2013V-1244 chromosome 1, complete sequence | *Vibrio parahaemolyticus* | 4045 | 4045 | 100% | 0 | 99.46 | 3276195 | CP046782.1 |
| *Vibrio parahaemolyticus* strain 2013V-1136 chromosome 1, complete sequence | *Vibrio parahaemolyticus* | 4045 | 4045 | 100% | 0 | 99.46 | 3397332 | CP046785.1 |
| *Vibrio parahaemolyticus* strain 2013V-1181 chromosome 1, complete sequence | *Vibrio parahaemolyticus* | 4045 | 4045 | 100% | 0 | 99.46 | 3418724 | CP046783.1 |
| *Vibrio parahaemolyticus* strain AM43962 chromosome 1, complete sequence | *Vibrio parahaemolyticus* | 4045 | 4045 | 100% | 0 | 99.46 | 3297364 | CP046776.1 |
| *Vibrio parahaemolyticus* strain FORC_072 chromosome 1, complete sequence | *Vibrio parahaemolyticus* | 4045 | 4045 | 100% | 0 | 99.46 | 3402675 | CP023472.1 |
| *Vibrio parahaemolyticus* strain MAVP-Q chromosome 1, complete sequence | *Vibrio parahaemolyticus* | 4045 | 4045 | 100% | 0 | 99.46 | 3369175 | CP022473.1 |
| *Vibrio parahaemolyticus* strain MAVP-Q chromosome 1, complete sequence | *Vibrio parahaemolyticus* | 4045 | 4045 | 100% | 0 | 99.46 | 3369172 | CP011884.1 |
| *Vibrio parahaemolyticus* strain 23EBVib0155 chromosome 1, complete sequence | *Vibrio parahaemolyticus* | 4045 | 4045 | 100% | 0 | 99.46 | 3324308 | CP128370.1 |
| *Vibrio parahaemolyticus* strain Vp2015094 chromosome 1, complete sequence | *Vibrio parahaemolyticus* | 4045 | 4045 | 100% | 0 | 99.46 | 3351532 | CP080478.1 |
| *Vibrio parahaemolyticus* strain vp-HL-202005 chromosome 1, complete sequence | *Vibrio parahaemolyticus* | 4045 | 4045 | 100% | 0 | 99.46 | 3528831 | CP114194.1 |
| *Vibrio parahaemolyticus* strain DHO76 chromosome I, complete sequence | *Vibrio parahaemolyticus* | 4045 | 4045 | 100% | 0 | 99.46 | 3277821 | CP066246.1 |
| *Vibrio parahaemolyticus* strain 2010V-1106 chromosome 1, complete sequence | *Vibrio parahaemolyticus* | 4039 | 4039 | 100% | 0 | 99.42 | 3311269 | CP046828.1 |
| *Vibrio parahaemolyticus* strain 2014V-1125 chromosome 1, complete sequence | *Vibrio parahaemolyticus* | 4039 | 4039 | 100% | 0 | 99.42 | 3323757 | CP046778.1 |
| *Vibrio parahaemolyticus* strain 2013V-1146 chromosome 1, complete sequence | *Vibrio parahaemolyticus* | 4039 | 4039 | 100% | 0 | 99.42 | 3343366 | CP046808.1 |
| *Vibrio parahaemolyticus* strain 2014V-1066 chromosome 1, complete sequence | *Vibrio parahaemolyticus* | 4039 | 4039 | 100% | 0 | 99.42 | 3323763 | CP046779.1 |
| *Vibrio parahaemolyticus* strain 2015AW-0174 chromosome 1, complete sequence | *Vibrio parahaemolyticus* | 4039 | 4039 | 100% | 0 | 99.42 | 3326911 | CP046754.1 |
| *Vibrio parahaemolyticus* 10329 chromosome 1, complete sequence | *Vibrio parahaemolyticus* 10329 | 4039 | 4039 | 100% | 0 | 99.42 | 3311004 | CP045794.1 |
| *Vibrio parahaemolyticus* strain FDAARGOS_662 chromosome 2, complete sequence | *Vibrio parahaemolyticus* | 4039 | 4039 | 100% | 0 | 99.42 | 3320553 | CP044071.1 |
| *Vibrio parahaemolyticus* strain FDAARGOS_51 chromosome 1, complete sequence | *Vibrio parahaemolyticus* | 4039 | 4039 | 100% | 0 | 99.42 | 3316147 | CP026041.1 |
| *Vibrio parahaemolyticus* strain MAVP-26 chromosome 1, complete sequence | *Vibrio parahaemolyticus* | 4039 | 4039 | 100% | 0 | 99.42 | 3360050 | CP023248.1 |
| *Vibrio parahaemolyticus* strain 17-VB00214 chromosome 1, complete sequence | *Vibrio parahaemolyticus* | 4039 | 4039 | 100% | 0 | 99.42 | 3382905 | CP062153.1 |
| *Vibrio parahaemolyticus* strain 19-VB00998 chromosome 1, complete sequence | *Vibrio parahaemolyticus* | 4039 | 4039 | 100% | 0 | 99.42 | 3421970 | CP062150.1 |
| *Vibrio parahaemolyticus* strain VP157 chromosome I, complete sequence | *Vibrio parahaemolyticus* | 4039 | 4039 | 100% | 0 | 99.42 | 3530958 | CP068641.1 |
| *Vibrio parahaemolyticus* strain S107-1 chromosome 1, complete sequence | *Vibrio parahaemolyticus* | 4037 | 4037 | 100% | 0 | 99.42 | 3445421 | CP028481.1 |
| *Vibrio parahaemolyticus* strain 2012AW-0353 chromosome 1, complete sequence | *Vibrio parahaemolyticus* | 4034 | 4034 | 100% | 0 | 99.37 | 3332037 | CP046763.1 |
| *Vibrio parahaemolyticus* strain MAVP-R chromosome 1, complete sequence | *Vibrio parahaemolyticus* | 4034 | 4034 | 100% | 0 | 99.37 | 3468665 | CP022552.2 |
| *Vibrio parahaemolyticus* strain HZ-52 chromosome I, complete sequence | *Vibrio parahaemolyticus* | 4034 | 4034 | 100% | 0 | 99.37 | 3471189 | CP047985.1 |
| *Vibrio parahaemolyticus* strain FORC_022 chromosome 1, complete sequence | *Vibrio parahaemolyticus* | 4028 | 4028 | 100% | 0 | 99.33 | 3358191 | CP013248.1 |
| *Vibrio parahaemolyticus* strain 16-VB00198 chromosome 1, complete sequence | *Vibrio parahaemolyticus* | 3962 | 3962 | 100% | 0 | 98.79 | 3355318 | CP097355.1 |
| *Vibrio parahaemolyticus* strain FDAARGOS_667 chromosome 1, complete sequence | *Vibrio parahaemolyticus* | 3895 | 3895 | 100% | 0 | 98.25 | 3260755 | CP044062.1 |
| *Vibrio parahaemolyticus* O1:Kuk str. FDA_R31 chromosome I, complete sequence | *Vibrio parahaemolyticus* O1:Kuk str. FDA_R31 | 3895 | 3895 | 100% | 0 | 98.25 | 3362228 | CP006004.1 |
| *Vibrio parahaemolyticus* strain XMO116 chromosome I, complete sequence | *Vibrio parahaemolyticus* | 3890 | 3890 | 100% | 0 | 98.2 | 3517946 | CP064041.1 |
| *Vibrio parahaemolyticus* strain XMM117 chromosome I, complete sequence | *Vibrio parahaemolyticus* | 3884 | 3884 | 100% | 0 | 98.16 | 3518178 | CP064037.1 |
| *Vibrio* *alginolyticus* strain XWV9 chromosome 1, complete sequence | *Vibrio* *alginolyticus* | 3851 | 3851 | 100% | 0 | 97.89 | 3352833 | CP082319.1 |
| *Vibrio parahaemolyticus* strain 2012AW-0154 chromosome 1, complete sequence | *Vibrio parahaemolyticus* | 3712 | 3712 | 100% | 0 | 96.77 | 3639425 | CP035701.1 |
| *Vibrio parahaemolyticus* strain 2012V-1165 chromosome 1 | *Vibrio parahaemolyticus* | 3607 | 3607 | 100% | 0 | 95.91 | 3411422 | CP051111.1 |
| *Vibrio navarrensis* strain 20-VB00237 chromosome 2, complete sequence | *Vibrio navarrensis* | 2060 | 2060 | 99% | 0 | 83.42 | 1395396 | CP065218.1 |
| *Vibrio natriegens* strain CCUG 16373 chromosome 2, complete sequence | *Vibrio natriegens* | 1893 | 1893 | 99% | 0 | 82.07 | 2050970 | CP016350.1 |
| *Vibrio natriegens* strain PWH3a chromosome 2, complete sequence | *Vibrio natriegens* | 1893 | 1893 | 99% | 0 | 82.1 | 1941516 | CP107283.1 |
| *Vibrio natriegens* strain WPAGA4 chromosome 2, complete sequence | *Vibrio natriegens* | 1893 | 1893 | 99% | 0 | 82.06 | 1994952 | CP094881.1 |
| *Vibrio natriegens* NBRC 15636 = ATCC 14048 = DSM 759 chromosome 2, complete sequence | *Vibrio natriegens* NBRC 15636 = ATCC 14048 = DSM 759 | 1877 | 1877 | 99% | 0 | 81.92 | 1927130 | CP009978.1 |
| *Vibrio natriegens* NBRC 15636 = ATCC 14048 = DSM 759 strain ATCC 14048 chromosome 2, complete sequence | *Vibrio natriegens* NBRC 15636 = ATCC 14048 = DSM 759 | 1877 | 1877 | 99% | 0 | 81.92 | 1927156 | CP016346.1 |
| *Vibrio natriegens* strain 14048 chromosome 2, complete sequence | *Vibrio natriegens* | 1877 | 1877 | 99% | 0 | 81.92 | 1927108 | CP101906.1 |
| *Vibrio* sp. dhg chromosome 2, complete sequence | *Vibrio* sp. dhg | 1871 | 1871 | 99% | 0 | 81.9 | 1995515 | CP028944.1 |
| *Vibrio* sp. EJY3 chromosome 2, complete sequence | *Vibrio* sp. EJY3 | 1871 | 1871 | 99% | 0 | 81.88 | 1974339 | CP003242.1 |
| *Vibrio natriegens* strain CCUG 16371 chromosome 2, complete sequence | *Vibrio natriegens* | 1855 | 1855 | 99% | 0 | 81.75 | 1849962 | CP016348.1 |
| *Vibrio natriegens* strain CCUG 16374 chromosome 2, complete sequence | *Vibrio natriegens* | 1849 | 1849 | 99% | 0 | 81.71 | 2044646 | CP016352.1 |
| *Vibrio* *diabolicus* strain FDAARGOS_96 chromosome 2, complete sequence | *Vibrio* *diabolicus* | 1517 | 1517 | 99% | 0 | 79.06 | 1773503 | CP014095.1 |
| *Vibrio* sp. STUT-A16 DNA, chromosome 2, complete sequence | *Vibrio* sp. STUT-A16 | 1517 | 1517 | 99% | 0 | 79.04 | 1881063 | AP026766.1 |
| *Vibrio* *diabolicus* strain FA3 chromosome 2, complete sequence | *Vibrio* *diabolicus* | 1511 | 1511 | 99% | 0 | 79.03 | 1852366 | CP042452.1 |
| *Vibrio* *diabolicus* strain FDAARGOS_105 chromosome 2, complete sequence | *Vibrio* *diabolicus* | 1511 | 1511 | 99% | 0 | 79.02 | 2032660 | CP014037.1 |
| *Vibrio* *diabolicus* strain Colony272 chromosome 2 | *Vibrio* *diabolicus* | 1506 | 1506 | 99% | 0 | 78.98 | 1773503 | CP078739.1 |
| *Vibrio* *alginolyticus* YM4 DNA, chromosome 2, complete sequence | *Vibrio* *alginolyticus* | 1500 | 1500 | 99% | 0 | 78.93 | 1759327 | AP022866.1 |
| *Vibrio* *alginolyticus* YM19 DNA, chromosome 2, complete sequence | *Vibrio* *alginolyticus* | 1500 | 1500 | 99% | 0 | 78.93 | 1759327 | AP022864.1 |
| *Vibrio* *alginolyticus* VIO5 DNA, chromosome 2, complete sequence | *Vibrio* *alginolyticus* | 1500 | 1500 | 99% | 0 | 78.93 | 1759333 | AP022862.1 |
| *Vibrio* *alginolyticus* 138-2 DNA, chromosome 2, complete sequence | *Vibrio* *alginolyticus* | 1500 | 1500 | 99% | 0 | 78.93 | 1759333 | AP022860.1 |
| *Vibrio* *diabolicus* strain NV27 chromosome 2, complete sequence | *Vibrio* *diabolicus* | 1500 | 1500 | 99% | 0 | 78.93 | 1794367 | CP085844.1 |
| *Vibrio* *diabolicus* strain SLV18 chromosome 2, complete sequence | *Vibrio* *diabolicus* | 1495 | 1495 | 99% | 0 | 78.88 | 1769354 | CP069197.1 |
| *Vibrio* antiquarius strain EX25 chromosome 2, complete sequence | *Vibrio* antiquarius | 1495 | 1495 | 99% | 0 | 78.89 | 1829445 | CP001806.1 |
| *Vibrio* *diabolicus* strain LMG 3418 chromosome 2, complete sequence | *Vibrio* *diabolicus* | 1478 | 1478 | 99% | 0 | 78.74 | 1875198 | CP014133.1 |
| *Vibrio* *alginolyticus* strain AUSMDU00064140 chromosome 2, complete sequence | *Vibrio* *alginolyticus* | 1478 | 1478 | 99% | 0 | 78.73 | 1886538 | CP110671.1 |
| *Vibrio* *diabolicus* strain R-31 chromosome 2 | *Vibrio* *diabolicus* | 1478 | 1478 | 99% | 0 | 78.75 | 1817616 | CP063378.1 |
| *Vibrio* *alginolyticus* strain LHF01 chromosome 2, complete sequence | *Vibrio* *alginolyticus* | 1461 | 1461 | 97% | 0 | 78.9 | 1874090 | CP087877.1 |
| *Staphylococcus capitis* strain FDAARGOS_753 chromosome, complete genome | *Staphylococcus capitis* | 65.8 | 65.8 | 1% | 5.00E-05 | 97.37 | 2467666 | CP053957.1 |
| *Staphylococcus capitis* strain CCSM0123 chromosome, complete genome | *Staphylococcus capitis* | 65.8 | 65.8 | 1% | 5.00E-05 | 97.37 | 2453862 | CP110784.1 |
| *Staphylococcus capitis* subsp. capitis strain DSM 20326 chromosome, complete genome | *Staphylococcus capitis* subsp. capitis | 65.8 | 65.8 | 1% | 5.00E-05 | 97.37 | 2443707 | CP103049.1 |
| *Deefgea tanakiae* strain D25 chromosome, complete genome | *Deefgea tanakiae* | 63.9 | 63.9 | 2% | 2.00E-04 | 87.5 | 3557673 | CP081150.1 |
| *Paenibacillus amylolyticus* strain Sample9_7 chromosome, complete genome | *Paenibacillus amylolyticus* | 60.2 | 60.2 | 2% | 0.002 | 89.36 | 6824844 | CP121451.1 |
| *Granulicatella adiacens* strain KHUD_009 chromosome, complete genome | *Granulicatella adiacens* | 56.5 | 56.5 | 1% | 0.028 | 100 | 1949834 | CP106751.1 |
| *Granulicatella adiacens* ATCC 49175 chromosome, complete genome | *Granulicatella adiacens* ATCC 49175 | 56.5 | 56.5 | 1% | 0.028 | 100 | 1965422 | CP102283.1 |
| *Granulicatella adiacens* strain FDAARGOS_1477 chromosome, complete genome | *Granulicatella adiacens* | 56.5 | 56.5 | 1% | 0.028 | 100 | 1965420 | CP082858.1 |
